# Rapid Seismic Evaluation of Continuous Girder Bridges with Localized Plastic Hinges

**DOI:** 10.3390/s22166311

**Published:** 2022-08-22

**Authors:** Zhaolan Wei, Mengting Lv, Minghui Shen, Haijun Wang, Qixuan You, Kai Hu, Shaomin Jia

**Affiliations:** School of Civil Engineering, Sichuan Agricultural University, Dujiangyan 611830, China

**Keywords:** bridge vibration, localized plasticity, nonlinear restoring forces, nonlinear stochastic vibration, plastic hinge

## Abstract

In seismic assessment of continuous girder bridges, plastic hinges form in bridge piers to dissipate seismic energy through nonlinear restoring forces. Considering temporal and spatial variations of ground motions, seismic evaluation of the bridges involves nonlinear stochastic vibration and expensive computation. This paper presents an approach to significantly increase the efficiency of seismic evaluation for continuous girder bridges with plastic hinges. The proposed approach converts nonlinear motion equations into quasi-linear state equations, solves the equations using an explicit time-domain dimension-reduced iterative method, and incorporates a stochastic sampling method to statistically analyze the seismic response of bridges under earthquake excitation. Taking a 3 × 30 m continuous girder bridge as an example, fiber beam-column elements are used to simulate the elastic–plastic components of the continuous girder bridge, and the elastic–plastic time history analysis of the continuous girder bridge under non-uniform seismic excitation is carried out. Results show that the computation time is only 5% of the time of the nonlinear time history approach while retaining the accuracy. This study advances the capability of rapid seismic assessment and design for bridges with localized nonlinear behaviors such as plastic hinges.

## 1. Introduction

Seismic evaluation and design of bridges play essential roles in improving resilience of life-line infrastructure. Continuous girder bridges are one of the most widely used types of bridges in highway and railway applications due to their advantages such as convenience of construction, high mechanical performance, and low cost. Continuous girder bridges are often supported by reinforced concrete piers. In seismic design of bridges, bridge piers are strategically designed to generate plastic hinges under high-intensity ground motions, to protect bridges from catastrophic collapse [1]. Typically, a plastic hinge is formed at the bottom section of a reinforced concrete column under earthquake excitation [2]. The plastic hinge possesses nonlinear restoring forces that dissipate seismic energy through yielding of steel bars and cracking and crushing of concrete, as well as interfacial slippage between steel bar and concrete [3]. Energy dissipation thus mitigates damage in the other parts of the bridge and facilitates post-earthquake condition assessment and repairing of damaged bridges [4]. Formation of a plastic hinge also changes the structural stiffness and natural frequencies, thus altering the structural response in an earthquake. Seismic evaluation of bridges with plastic hinges involves nonlinear structural dynamics. In addition, ground motions involve random temporal and spatial variations [5]. Therefore, seismic evaluation and design of bridges involves nonlinear structural dynamics and random earthquake excitations.

Currently, seismic evaluation and design of bridges mainly uses a response spectrum method [6], which does not fully consider the nonlinear structural dynamics and random excitations. It is desired to develop effective approaches to perform nonlinear stochastic dynamic analysis. To this end, different approaches were proposed, such as nonlinear dynamic analysis [7,8,9], perturbation analysis [10], and statistical linearization [11]. Nonlinear dynamic analysis of bridges considers nonlinear material properties and the formation of plastic hinges, but random earthquake excitations are not considered. Statistical or equivalent linearization and perturbation analysis provide a general method to address random vibration problems [12], but many of those approaches are inconvenient to apply in real practice. In general, many equivalent linear systems are established at different time instants, inefficient for bridges subjected to spatially varying stochastic ground motions. Recently, a tail-equivalent linearization approach has been proposed to analyze nonlinear structural dynamics under spatially varying stochastic ground motions and shows good promise in the context of nonlinear stochastic dynamic analysis [13,14]. Simulation of random processes offers another promising avenue to address nonlinear stochastic dynamic analysis [15]. However, as a random sampling method in nature is time consuming since it involves analysis of many samples. To improve the computational efficiency, explicit time-domain solutions were proposed to reduce the time consumption in the analysis of each sample [16]. These related advances offer new opportunities for improving the computational efficiency of nonlinear stochastic dynamic analysis with spatially varying ground motions.

Based on the above important development, this research aims to develop an approach to perform nonlinear stochastic dynamic analysis for bridges subjected to spatially varying ground motions with high efficiency. The proposed approach takes advantage of the feature of localized plastic hinges to improve the computation efficiency because the nonlinearity is only considered locally rather than globally, different from conventional nonlinear dynamic analysis [7]. The proposed approach converts nonlinear motion equations into quasi-linear state equations, adopts explicit time-domain analysis and dimension reduction methods to solve the equations, and incorporates a stochastic sampling method to statistically analyze the seismic response of bridges under random earthquake excitations [16,17]. The proposed approach is implemented into a representative case study on a three-span continuous girder bridge with two reinforced concrete piers under spatially varying stochastic ground motions. Nonlinear restoring forces of plastic hingers in the piers are considered. The explicit analysis method is expected to achieve higher efficiency and convergence performance than the implicit analysis methods in previous research. The novelty and contribution of this research is mainly reflected by the development of the approach to significantly improve the efficiency of nonlinear stochastic dynamic analysis of bridges under spatially varying ground motions. The approach is expected to greatly advance the capability of rapid seismic evaluation of bridges. The remainder of the paper includes three main sections: Section 2 elaborates the approach for nonlinear stochastic dynamic analysis of continuous girder bridges. Section 3 discusses the case study for implementation of the proposed approach. Section 4 summarizes the conclusions of this research.

## 2. Methodology

**Figure 1:**
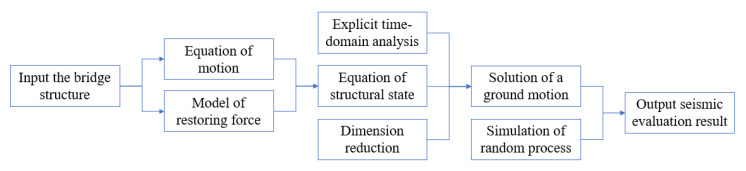
Flowchart of the proposed approach to perform nonlinear stochastic dynamic analysis.

The flowchart of the proposed approach is shown in Figure 1. With an input bridge, the mass, damping, and stiffness matrices are determined, thus obtaining the equation of motion. For a specific bridge, the restoring forces of plastic hinges are determined. With the equation of motion and restoring forces, the implicit equation of motion is converted to explicit expressions based on the equation of state. The explicit equations are solved through explicit time-domain analysis and dimension reduction. The solution of the equations represents the solution of the given bridge under a specific ground motion. To evaluate seismic responses of the bridge under random earthquake excitations, more ground motions are considered based on simulation of random processes, which generates different samples of ground motions. For each sample of ground motions, the nonlinear dynamic response of the bridge is analyzed using the proposed approach. Finally, the statistical result of the seismic response of the bridge is delivered as the output.

The detailed techniques that are incorporated in the proposed approach are elaborated in the following subsections. Section 2.1 presents the equation of motion of continuous girder bridges. Section 2.2 introduces a nonlinear restoring force model of plastic hinges in reinforced concrete piers. Section 2.3 details the derivation of the equation of state for explicit time-domain analysis. Section 2.4 elaborates the dimension reduction method. Section 2.5 discusses the simulation of random process.

### 2.1. Equation of Motion

**Figure 2:**
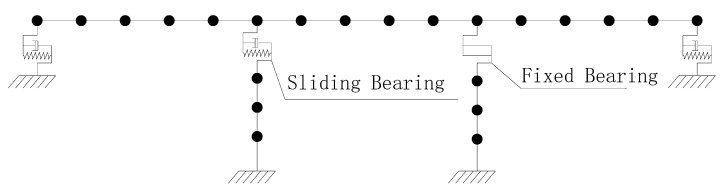
Diagram of continuous girder bridge finite element discrete model.

A general model of continuous girder bridges is illustrated in Figure 2. A bridge is divided into a number of elements representing the girders, supports, and piers. The numbers of free nodes, supporting nodes, and piers are denoted by n, m, and l, respectively.

According to the theory of structural dynamics [17], the equation of motion is expressed as:

(1) $$\begin{array}{l} \left[\begin{array}{cc} M_{ss} & 0\\ 0 & M_{bb} \end{array}\right]\left\{\begin{array}{c} {\ddot{X}}_{s}\\ {\ddot{X}}_{b} \end{array}\right\}+\left[\begin{array}{cc} C_{ss} & 0\\ 0 & C_{bb} \end{array}\right]\left\{\begin{array}{c} {\dot{X}}_{s}\\ {\dot{X}}_{b} \end{array}\right\}+\\ \left[\begin{array}{cc} K_{ss} & K_{sb}\\ K_{bs} & K_{bb} \end{array}\right]\left\{\begin{array}{c} X_{s}\\ X_{b} \end{array}\right\}+\left[\begin{array}{cc} D & 0\\ 0 & 0 \end{array}\right]\left\{\begin{array}{c} F_{r}(X_{l})\\ 0 \end{array}\right\}=\left\{\begin{array}{c} 0\\ P_{b} \end{array}\right\} \end{array}$$

where ***X**_s_* is the displacement of free nodes; ***X**_b_* is the displacement of supporting nodes; ***X**_l_* is the relative displacement of the nodes of the plastic hinges in the piers; *D* is the indicator matrix of the position of the restoring force in the plastic hinge region; ***F**_r_*(***X**_l_*) is the horizontal restoring force column vector of central plastic hinges; ***P**_b_* is the force due to ground motions; ***M***, ***C***, and ***K*** are the mass, damping, and stiffness matrices, respectively; and subscripts s and b represent free nodes and supporting nodes, respectively.

According to Equation (1), the following equation can be derived by rewriting Equation (1).

(2) $$M_{ss}{\ddot{X}}_{s}+C_{ss}{\dot{X}}_{s}+K_{ss}X_{s}\approx -K_{sb}X_{b}-DF_{r}(X_{l}),$$

Equation (2) is rewritten according to Equation (1), and all parameters are the same as that in Equation (1).

### 2.2. Restoring Force Model of Plastic Hinges

Different types of restoring force models were proposed in the literature [18], such as the bilinear model, modified bilinear model, equivalent bilinear model, hysteresis model, and Bouc–Wen model. The Bouc–Wen model is numerically tractable and has been extensively used for modeling hysteretic systems [19], showing desired performance in simulating dynamic behaviors of plastic hinges. The Bouc–Wen model contains components from a viscous damper, a spring, and a hysteretic component, expressed as:

(3) $$F_{r}=C_{b}\dot{u}+\alpha \frac{F_{y}}{D_{y}}u+\left(1-\alpha \right)F_{y}Z,$$

(4) $$\dot{Z}=\frac{\gamma }{D_{y}}z|z\dot{u}|-\frac{\beta }{D_{y}}z^{2}\dot{u}+\frac{A}{D_{y}}\dot{u},$$

where *C_b_* is the generalized viscous damping ratio; *α* is the ratio of the generalized stiffness of the plastic hinge after it is yielded and the original stiffness; *F_y_* and *D_y_* are the generalized yielding force and generalized yielding displacement(curvature) of the plastic hinge, respectively; *u* and $\dot{u}$ are the generalized relative displacement and velocity of the plastic hinge; *z* is the hysteretic variable; and coefficients *γ*, *β*, and *A* are the model parameters depending on the shape of the hysteretic curve. In this research, coefficients *γ*, *β*, and *A* are equal to 0.5, 0.5, and 1.0, respectively.

### 2.3. Equation of State for Explicit Time-Domain Analysis

With the restoring force model of plastic hinges, Equation (2) can be rewritten as:

(5) $$M_{ss}{\ddot{X}}_{s}+\left(C_{ss}+C_{se}\right){\dot{X}}_{s}+\left(K_{ss}+K_{se}\right)X_{s}=-K_{sb}X_{b}-K_{h}Z,$$

where ***K**_se_*, ***K**_h_*, and ***C**_se_* are the equivalent stiffness, hysteretic stiffness, and equivalent damping matrices, respectively; and ***Z*** can be expressed as:

(6) $$\dot{Z}=g\left(t,U,Z(t)\right)\;U={\dot{X}}_{t}-{\dot{X}}_{b},$$

where ${\dot{X}}_{t}$ and ${\dot{X}}_{b}$ are the velocities of the top and bottom nodes of the plastic hinge, respectively.

To solve for the displacement (***X***) from Equation (5), it is necessary to know ***Z***. To solve for ***Z*** from Equation (6), it is necessary to know the velocities (${\dot{X}}_{t}$, ${\dot{X}}_{b}$). It is time consuming to directly solve the coupled equations expressed in the implicit form. This research proposes to convert the coupled equations into the state equation [20,21]:

(7) $$\dot{V}=HV+F(t),$$

(8) $$\begin{array}{l} V=\left\{\begin{array}{c} X_{s}\\ {\dot{X}}_{s} \end{array}\right\}\;F(t)=W\bar{F}(t)=\left\{\begin{array}{c} 0\\ M_{ss}{}^{-1} \end{array}\right\}\bar{F}(t)\\ H=\left[\begin{array}{cc} 0 & I\\ -M_{ss}{}^{-1}\left(K_{ss}+K_{se}\right) & -M_{ss}^{-1}\left(C_{ss}+C_{se}\right) \end{array}\right] \end{array}$$

(9) $$\bar{F}(t)=-K_{sb}X_{b}-K_{h}Z,$$

The solutions of Equations (7)–(9) are expressed in Equations (10) and (11) [20], when $\bar{F}(t)$ is linear in the time interval Δ*t* and the initial control is $V_{0}(t_{0})=0$.

(10) $$V_{i}=\sum_{j=0}^{i}A_{i,j}\bar{F}(t_{j}),$$

(11) $$\bar{F}(t_{i})=-K_{sb}X_{b}(t_{i})-K_{h}Z_{i},$$

where ***A***(*i*,*j*) are the coefficient matrices dependent on *i*, and *i* = 1, 2, …, *p*. When *i* = 1, ***A***_1,0_ = ***S***_1_, and ***A***_1,1_ = ***S***_2_. When *i* = 2, ***A***_2,0_ = ***TA***_1,0_, ***A***_2,1_ = ***TS***_2_ + ***S***_1_, and ***A***_2,2_ = ***A***_1,1_. When *i* ≥ 3, ***A***_(*i,0*)_ = ***TA***_(*i−1*,0)_, ***A***_(*i*,1)_ =***TA***_(*i*−1,1)_, and ***A***_(*i,j*)_ = ***A***_(*i*−1,*j*−1)_. ***T*** is the exponential matrix. ***S*_1_** and ***S*_2_** are shown as:

(12) $$S_{1}=\frac{\left(I-T\right)W}{H^{2}\Delta t}+\frac{TW}{H},$$

(13) $$S_{2}=\frac{\left(T-1\right)W}{H^{2}\Delta t}-\frac{W}{H},$$

Based on Equations (10)–(13), the coefficient matrices of each time instant are determined through iterations, once ***A***_(*i*,0)_ and ***A***_(*i*,0)_ are determined, thus solving the state equation. This study incorporates the Runge–Kutta method to improve computation precision in the iterations [22,23]. The recursion formula is expressed as:

(14) $$Z_{i}\left(s_{k+1}\right)\approx Z_{i}\left(s_{k}\right)+\frac{1}{6}\left(k_{1}+2k_{2}+2k_{3}+k_{4}\right),$$

(15) $$k_{1}=hg(t_{i-1},\;Z_{i-1}),$$

(16) $$k_{2}=hg(t_{i-1}+\frac{h}{2},\;Z_{i-1}+\frac{k_{1}}{2}),$$

(17) $$k_{3}=hg(t_{i-1}+\frac{h}{2},\;Z_{i-1}+\frac{k_{2}}{2}),$$

(18) $$k_{4}=hg(t_{i-1}+h,\;Z_{i-1}+k_{3}),$$

where *s_k_* = *kh*, *k* = 0, 1, …, *n* − 1; and *h* = Δ*t*/*n*. When *k* = 0, ***Z****_i_* (*s_k_*) = ***Z***_(*i*−1)_. When *k* = *n*, ***Z****_i_*(*s_k_*) = ***Z****_i_*.

**Figure 3:**
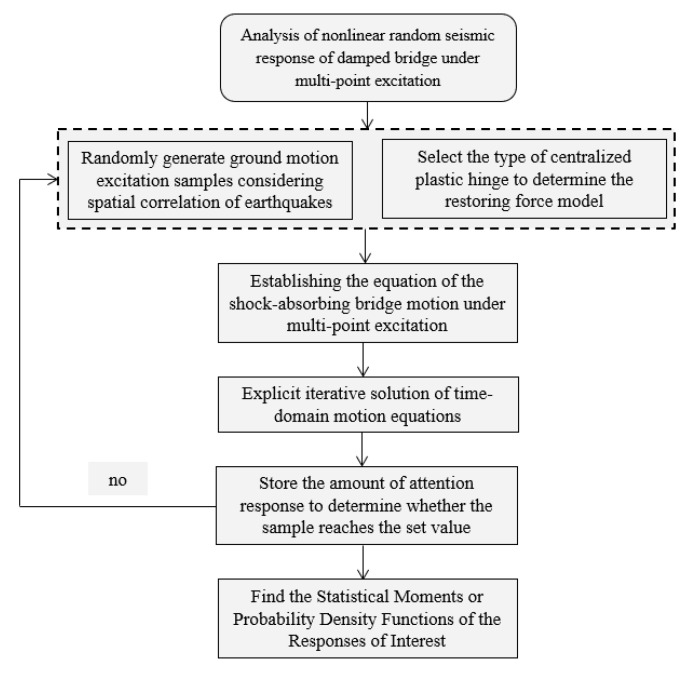
Flowchart of the solving process for the hysteretic variable through iterative calculations.

Then, the structural dynamic response and hysteretic variable can be determined following the flowchart in Figure 3. Given an initial value of the hysteretic variable ***Z****_i_*, the corresponding state ***V****_i_* can be solved using Equations (10) and (11) and Equations (12) and (13), in turn obtaining (${\dot{X}}_{t}$) and (${\dot{X}}_{b}$), which are plugged into Equations (14)–(18) to obtain the updated ***Z****_i_*. This process is continued until the termination criterion is satisfied. In this research, the adopted criterion is expressed as:

(19) $$\frac{\left\|V_{i,j}-V_{i,j-1}\right\|}{V_{i,j}}\leq \varepsilon ,$$

where *ε* is the target precision; and operator ‖*‖ is the Euclidean norm.

The solving process illustrated in Figure 3 is formulated in Equations (24) and (25). According to Equations (24) and (25), an iteration is explicitly performed at each time instant. Once the coefficient matrix ***A***_(*i,i*)_ is generated, the matrix is retained and used in sequential calculations. In each time step, the calculation only involves $A_{i,i}F(t_{i})$ and $g\left({\dot{X}}_{t},\;{\dot{X}}_{b},\;Z_{i}\right)$. Therefore, the proposed approach is expected to achieve high computational efficiency. The performance of the proposed approach is tested through a case study, as elaborated in Section 3.

(20) $$\begin{array}{l} Z_{i-1,0}=Z_{i-1}\\ \bar{F}(t_{i})=-K_{sb}X_{b}(t_{i})-K_{h}Z_{i,j-1}\\ V_{i,j}=\sum_{j=0}^{i}A_{i,j}\bar{F}(t_{j})\\ Z_{i,j}=Z_{i}(s_{n}\_) \end{array}$$

### 2.4. Dimension Reduction

This research presents a dimension reduction technique to further improve the computational efficiency. It is noted that, in Equation (11), only one term contains the hysteretic variable ***Z****_i_*, and the other nodal forces are determined. The hysteretic variable is only related to the velocities of the two ends of each plastic hinge. Based on this understanding, ***V****_i_* is expressed as:

(21) $$V_{i}=\tilde{V_{p}}\left({\dot{X}}_{i,t}^{},{\dot{X}}_{i,b}^{}\right)+{\hat{V}}_{i}\left({\dot{X}}_{i,t}^{},{\dot{X}}_{i,b}^{}\right),$$

where $\tilde{V_{p}}\left({\dot{X}}_{i,t}^{},{\dot{X}}_{i,b}^{}\right)$ is directly associated with the nodes of the plastic hinges; and ${\hat{V}}_{i}\left({\dot{X}}_{i,t}^{},{\dot{X}}_{i,b}^{}\right)$ is not directly associated with the plastic hinges. Correspondingly, Equations (10) and (11) can be rewritten as:

(22) $$V_{i}=\sum_{j=0}^{p}\tilde{A_{p,J}}\bar{F}(t_{j})+\sum_{j=0}^{p}{\hat{A}}_{p,J}\bar{F}(t_{j}),$$

(23) $$\bar{F}(t_{i})=-K_{sb}X_{b}(t_{i})-K_{h}Z_{i},$$

According to Equations (14)–(18) and Equation (22), iterations are performed to calculate ***Z****_i_*, which is then plugged into Equation (23) to obtain the structural dynamic response. This solving process is the same as that in Section 2.3, but the number of equations is significantly reduced because the number of nodes involving plastic hinges in the piers is much smaller than the total number of nodes of the bridge.

### 2.5. Simulation of Random Processes

Based on the solving process of the equation of motion of the bridge under a single ground motion, simulation of random processes is incorporated to consider the stochastic variation of the ground motion [24,25]. Simulation of random processes generates samples of ground motions. The total number of ground motions is denoted as *N*. The *q*-th sample is denoted as ***F****_q(t)_*, and *q* = 1, 2, …, *N*. The nonlinear dynamic response of the bridge under the *q*-th sample of ground motion can be determined following the process elaborated in Section 2.1, Section 2.2, Section 2.3 and Section 2.4. It should be noted that, once the coefficient matrix is determined in the first ground motion, the coefficient matrix is sustained and directly used in calculations for the other ground motions without making any change, thus improving the computational efficiency.

After the dynamic responses of the bridge under the *N* ground motions are determined, the statistical data are obtained for each component of structural responses. For component *v_i_*, the mean and standard deviation are calculated as:

(24) $$E\left(v_{i}\right)=\frac{1}{N}\sum_{j=1}^{N}v_{ij},$$

(25) $$\sigma_{v_{i}}^{}=\sqrt{\frac{1}{N-1}\sum_{j=1}^{N}{\left[v_{ij}-E\left(v_{i}\right)\right]}^{2}},$$

## 3. Case Study

### 3.1. Bridge Description

**Figure 4:**
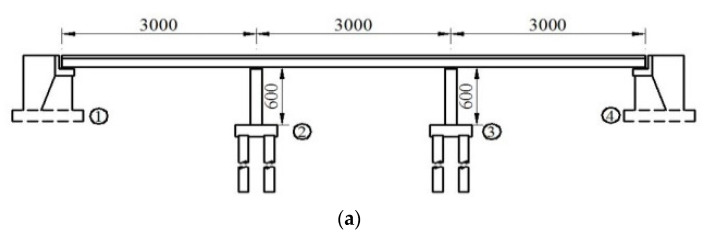

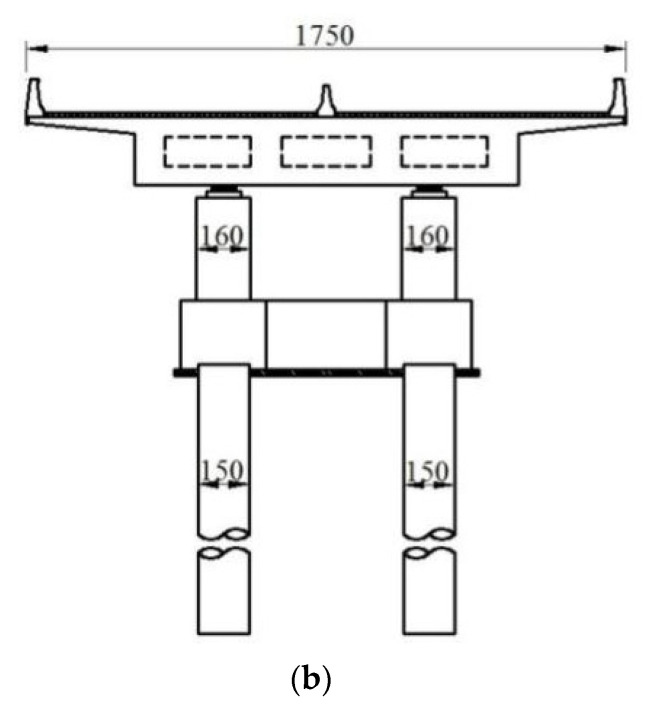
Illustration of the bridge (unit: cm): (**a**) longitudinal profile showing spans of bridge; and (**b**) the transverse profile showing the width of the bridge.

There is a 3 × 30 m prestressed concrete continuous girder bridge on a certain first-grade highway (the elevation layout is shown in Figure 4a). Its basic parameters are as follows. Bridge width is 17.5 m. The cross-section of the main girder, 2 m in height, adopts a single box with three cells. The double-column piers with circular solid sections are 6 m in height and 1.6 m in diameter, with its pile foundation 1.5 m in diameter. The bridge abutment is a U-shaped gravity one with an enlarged foundation. Fixed pot bearings are set on the No.② pier (denoted as *P*2), and movable bearings are set on the other piers or abutments. The overall cross-section layout of the bridge is shown in Figure 4b.

The bridge girders, piers, and foundations are made of concrete reinforced using deformed steel bars. The concrete grades of the bridge girders, piers, and foundations are C50, C40, and C30 respectively [26]. The material properties and geometrical properties of the bridge are listed in Table 1.

**Figure 5:**
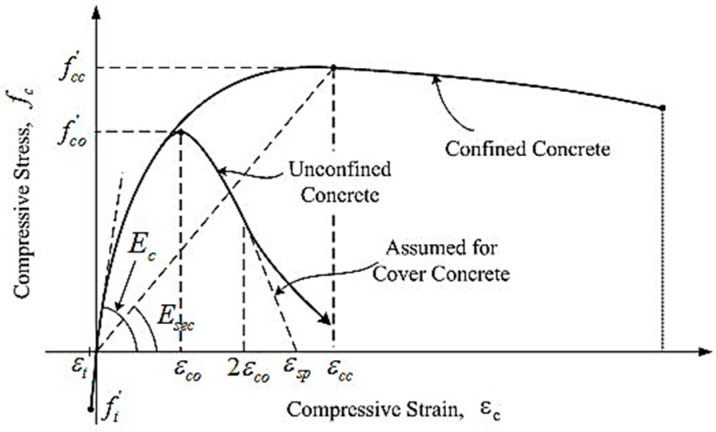
Schematic diagram of the Mander constitutive model.

**Figure 6:**
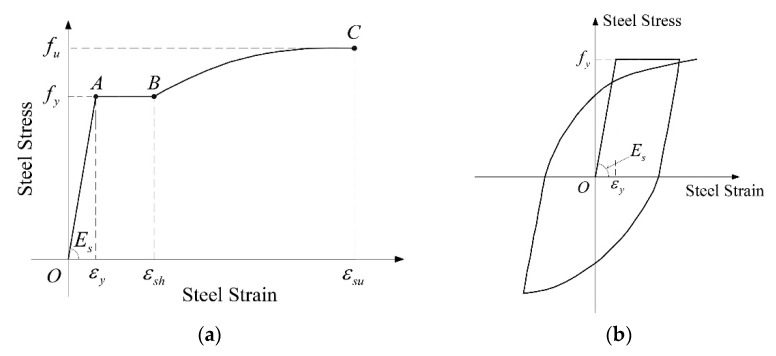
Park constitutive model diagram. (**a**) Stress–strain curve when loading. (**b**) Disposal strain curve, again when loading.

The whole bridge model is established with OpenSees. The section type of the model is selected as “fiber section”, and the element type as “element BeamWithHinges”, which can be used as the carrier of the centralized plastic hinge analysis. The non-confined concrete is divided into 2 parts in the radial direction and 18 parts in the circumferential direction. The confined concrete is divided into 4 parts in the radial direction and 18 parts in the circumference. The Mander model is adopted for the constitutive model of constrained and unconstrained concrete in this analysis, as shown in Figure 5. The constitutive model of the reinforcement adopts the Kent–Part model, which is suitable for repeated periodic loads, such as seismic load, as depicted in Figure 6. The parameters of concrete and steel bars calculated according to the model are shown in Table 2 and Table 3. The model constraints are handled as follows: the pier bottom is simulated by a consolidation mode, the connection between pier and beam by an ordinary elastic bearing, and the connection between abutment and beam by an ordinary constraint mode. After the above work, the parameters involved in the Bouc–Wen model are input, the output response index is defined, and then the ground motion parameters are defined. In this process, a large number of seismic loads are input, and finally the calculation and analysis are carried out. Since the focus is on the explicit iterative method applied to the rapid evaluation of seismic performance of continuous girder bridges, soil–structure interaction is not considered.

**Figure 7:**
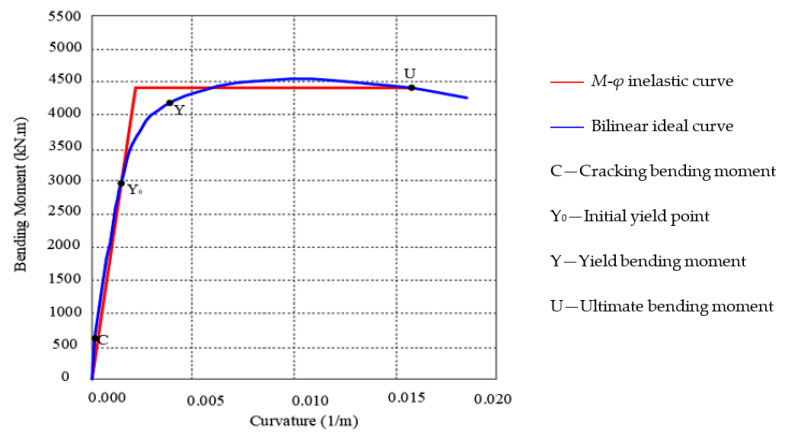
Plot of the moment–curvature relationship of the cross section of the bridge pier.

According to Clause 3.4.2 of Reference [27], for B-class bridges, the energy dissipation part is mainly located at the pier under the action of an earthquake, and the potential plastic hinge area of the double-column pier is at the pier bottom. In this paper, the plastic hinge is also set to form at the bottom of the pier. In accordance with the cross section and reinforcement, the moment–curvature relationship of the pier section is plotted in Figure 7. The yielding force is *F_y_* = 4169 kN/m. The initial stiffness is *K*_0_ = 4169 kN/m. After yielding occurs, the stiffness is *K_y_* = 41.69 kN/m.

The damping matrix of the bridge is determined using the Rayleigh damping model [28,29], and the mass and stiffness coefficients are equal to 0.26088 and 0.00073, respectively. In the Bouc–Wen model of the restoring force of plastic hinges, coefficients *γ*, *β*, and *A* are equal to 0.5, 0.5, and 1.0, respectively. The target precision in Equation (19) is $\varepsilon =10^{-5}$.

### 3.2. Power Spectrum Model of Ground Motions

In general, the soil conditions of different bridge piers are different. In this research, it is assumed that piers *P*1 and *P*2 are respectively supported by hard soil and medium soil, according to [30]. The propagation direction of the earthquake is from *P*1 to *P*2, both of which are subjected to the longitudinal correlated earthquake excitations. The Clough–Penzien model of ground motion [31,32], which has a double filtering feature, is adopted to describe the auto-power displacement spectrum of the time history of ground motion, expressed as:

(26) $$S_{kk}(\omega )=S_{0}\frac{\omega_{k}^{4}+4\zeta_{k}^{2}\omega_{k}^{2}\omega^{2}}{{\left(\omega_{k}^{2}-\omega^{2}\right)}^{2}+4\zeta_{k}^{2}\omega_{k}^{2}\omega^{2}}\times \frac{1}{{\left(\omega_{sk}^{2}-\omega^{2}\right)}^{2}+4\zeta_{sk}^{2}\omega_{sk}^{2}\omega^{2}},$$

where $S_{0}$ is the magnitude of spectrum, $S_{0}$ = 9.413 × 10^−3^ m^2^/s^3^, corresponding to an earthquake magnitude of *M*8 and an earthquake level of *E*2 [33]; $\omega_{k}$ and $\zeta_{k}$ are the natural frequency and damping ratio of soils, respectively; and $\omega_{sk}$ and $\zeta_{sk}$ are the frequency and damping parameters of the filter. The values of $\omega_{k}$, $\zeta_{k}$, $\omega_{sk}$, and $\zeta_{sk}$ are listed in Table 4.

### 3.3. Spatially Varying Excitation

In simulation of spatially varying earthquake excitations, this research considers the spatial correlation between ground motions, and the spatial correlation is described using the Luco–Wang model [34,35], as expressed in Equation (27):

(27) $$\left|\rho_{jk}(\omega ,d_{jk})\right|=\exp\left[-{\left(\frac{\alpha \omega d_{jk}}{v_{s}}\right)}^{2}\right],$$

where *ρ_jk_* is the coherence function of the stationary random processes $x_{j}(t)$ and $x_{k}(t)$ at the support points *j* and *k*; *ω* is the natural frequency; *d_jk_* is the projection of the horizontal distance between support points *j* and *k* along the direction of wave propagation; *α* is the correlation coefficient; and vs. is the propagation velocity of the earthquake wave. In this research, the values of *α* and vs. are 0.2 and 600 m/s, respectively.

Although the value of the cut-off frequency *ω_u_* of the multi-point ground motions has no influence on the proposed method in this article, it is still set as more than 5 times of the fundamental frequency according to Reference [33]. The duration of earthquake T should be several times longer than the fundamental period of the bridge. The frequency interval *dω* and the time interval *dt* are selected according to the Shannon sampling law [33]. Therefore, the cut-off frequency of the multi-point ground motions is $\omega_{u}=15\pi \;\mathrm{rad}/s$. The duration of the earthquake is *T* = 10 s, which is the total duration of earthquake excitation required for calculation and analysis. The frequency interval is $d\omega =\omega_{u}/N$, where the interval number *N* is equal to 300. The time interval is dt=0.01 s.

### 3.4. Results and Discussions

In order to verify the accuracy of the method proposed in this paper, 500 groups of ground motion samples are generated by using the ground motion simulation method mentioned in Reference [33] and the ground motion power spectrum parameters introduced in Section 3.2 and Section 3.3. Next, these ground motion samples are applied to the bridge described in Section 3.1. Then, the traditional *Newmark-β* method and the explicit time-domain dimension-reduced iterative method established in this paper are used to analyze the random seismic response of the bridge.

**Figure 8:**
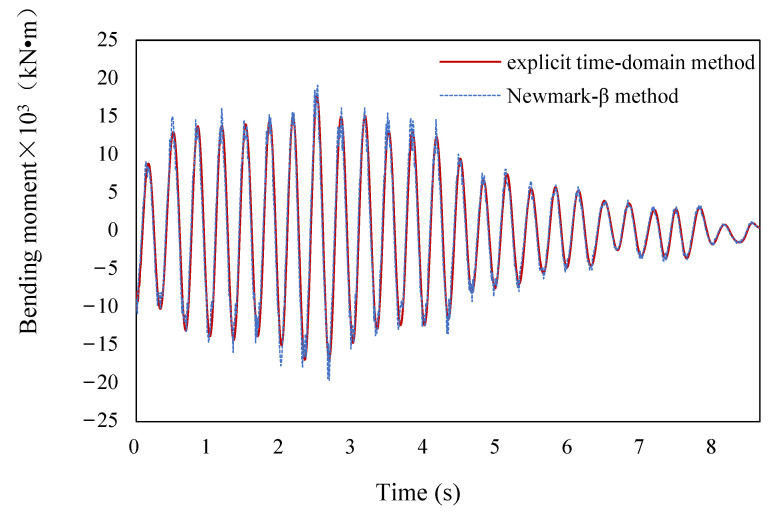
Time-history diagram of the bending moment at the bottom section of *P*2.

**Figure 9:**
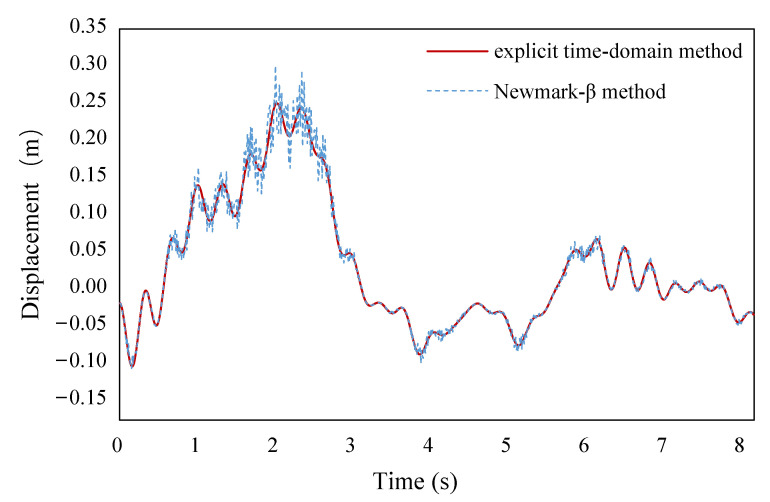
Displacement time-history diagram at the top section of *P*2.

When the ground motions are applied to the bridge, the calculation results are shown in the figures below. Figure 8 shows a time-history diagram of the bending moment at the bottom section of *P*2 where plastic hinges are formed. Figure 9 shows displacement time-history diagram at the top section of *P*2. It can be seen that the calculation results which are generated by the two methods mentioned above—the explicit time-domain dimension-reduced iterative method and the *Newmark-β* method—are basically consistent with each other. They are almost coincident at most moments, and the maximum error remains within 10% (the error is defined as the absolute value of the difference between the two methods divided by the calculated value of the *Newmark-β* iterative method), which verifies the accuracy of the explicit time-domain dimension-reduced iterative method in this paper. Throughout the analysis, the numerical stability of the explicit method is always checked. After several analyses, it is shown that the dimensionality reduction iteration has good stability and can be used to analyze local nonlinear random response problems.

**Figure 10:**
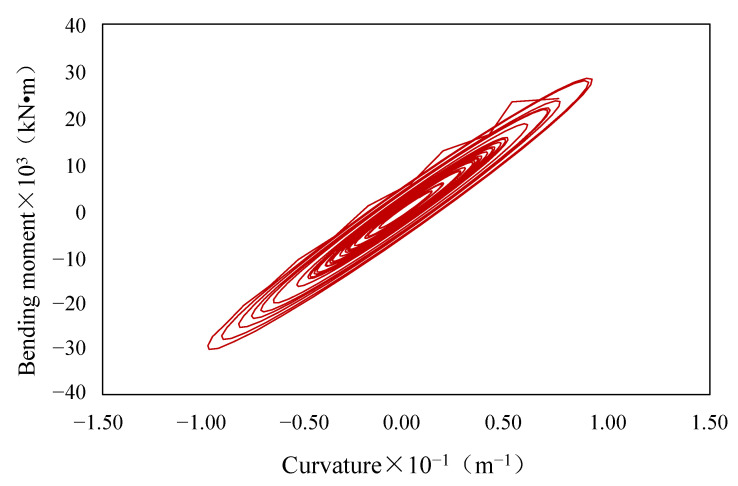
Bending moment–curvature diagram at the bottom section of *P*2.

In order to check the development of the plastic deformation of the pier section, the bending moment–curvature at the bottom section of *P*2 in Figure 10 is given. It can be concluded that, under non-uniform seismic excitation, the pier enters the elastic–plastic state and its stress behavior exhibits highly nonlinear characteristics. The method proposed in this paper is aimed at this characteristic.

Table 5 shows a time-consuming comparison of two methods, 250 s with the explicit time-domain dimension-reduced iterative method and 5000 s with the *Newmark-β* method. That is to say, the calculation and analysis time of the two methods for each seismic wave input is about 0.2 s and 10 s respectively. Obviously, the explicit time-domain dimension-reduced iterative method proposed in this paper has higher computational efficiency. This is mainly because this method only performs nonlinear dimension reduction iterative calculations on the elastic–plastic fiber elements of the pier column, which avoids the repeated inversion of the dynamic stiffness matrix and reduces the number of unknowns in the nonlinear equation system. Additionally, the traditional *Newmark-β* method needs to bundle all the degrees of freedom of the bridge together for iterative calculation. In addition, it can be seen from the derivation process in Section 2 that, since the responses have explicit expressions and the coefficient matrix only needs to be formed once, with the increase in the total number of degrees of freedom, the advantages of this method proposed in the paper are more obvious.

It should be pointed out that the calculation time of the explicit time-domain dimension-reduced iterative method mainly includes two parts. One part is used to construct the coefficient matrix in the explicit expression of dynamic response shown in Equations (10) and (11), which takes 215.9 s. The other part is used for the dimension-reduced iterative calculation, which only takes 34.1 s. However, the coefficient matrix only depends on the parameters of the structure itself, which only needs to be calculated once, and can be used repeatedly in all the sample analysis of the subsequent random simulation.

The calculation accuracy and efficiency of the explicit time-domain dimension-reduced iterative method for elastoplastic time-history analysis are verified, and the basic knowledge of probability theory can be further used to obtain the statistical values of each control section. Thus, the statistical values can be applied to evaluate its seismic performance quickly according to the corresponding bridge seismic design code, such as in reference [26].

**Figure 11:**
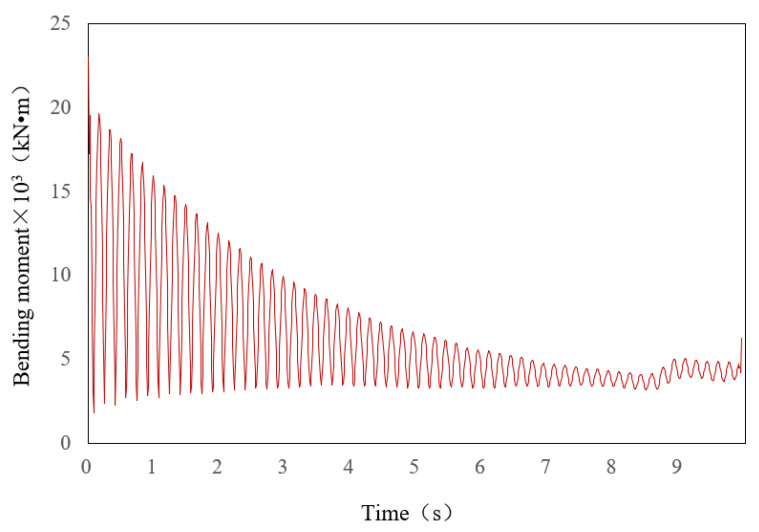
Standard deviation of the bending moment at the bottom section of *P*2.

The average, standard deviation, and maximum value of structural response are needed to evaluate the seismic performance of the bridge structure under random earthquake action. Table 6 shows the statistical values of bending moment at the bottom section of pier 2 under the excitation of 500 groups of displacement time history, the average value, the average value of the standard deviation, and the average value of its absolute maximum, which can be calculated by Equations (22)–(25). Taking the standard deviation time-history curve of the bending moment as an example, which is shown in Figure 11, the standard deviation value changes greatly at the beginning, but it becomes stable after a period of time. When the statistical values are obtained, the seismic performance evaluation of the bridge can be carried out.

## 4. Conclusions

This research presents an approach to evaluating the seismic responses of continuous girder bridges with plastic hinges formed at the bridge piers. The approach is developed to achieve high computational efficiency in the nonlinear stochastic dynamic analysis of bridges. The approach is implemented into a case study on a typical three-span continuous girder bridge with reinforced concrete piers. Based on the above investigations, the following conclusions are drawn:

(1) The proposed approach is accurate. The seismic performance evaluation of continuous girder bridges mainly focuses on the seismic performance of piers with fixed hinges. The seismic performance evaluation index is mainly based on pier top section displacement, pier bottom section bending moment–curvature, etc. The time history diagrams of pier top section displacement and the pier bottom section bending moment are consistent with the response characteristics of general dynamic problems, so the accuracy of the method can be known.
(2) The proposed approach significantly improves the computational efficiency. Once determined in the first round of analysis, the coefficient matrices in the equations are preserved throughout the analysis. Preservation of the coefficient matrices simplifies the computation process, thus improving computational efficiency. Such improvement is particularly relevant for seismic evaluation because the evaluation involves stochastic processes and requires repeated computations. Compared with the conventional nonlinear time history dynamic analysis performed, the computation time of the proposed approach is only 5%, and the maximum error of the displacement of the pier top section and the bending moment of the pier bottom section is within 10%. The high efficiency of the proposed approach is achieved by the combination of multiple techniques such as explicit time domain analysis using the state equations, the precision integration method, and the dimension reduction method.
(3) The proposed approach represents an explicit nonlinear dynamic analysis method. The bending moment–curvature diagram shows an oval shape, which indicates that the central plastic hinge area of the continuous girder bridge pier has significant nonlinear characteristics and can dissipate certain ground motion energy. Under the current Chinese seismic design code, these problems are typical local nonlinear problems (nonlinearities occur near the pier bottom or pier top section with fixed hinges), and the explicit iterative dimension reduction method can be used to ensure the high efficiency of seismic performance evaluation. Compared to the conventional methods, which implicitly solve the equations of motion based on iterative computation involving matrix inversion, the proposed explicit method is expected to have better convergence performance. It is worth noting that the dimension reduction utilizes the unique feature of localized plastic hinges. The dimension reduction method is likely applicable to other nonlinear stochastic dynamic problems involving local plasticity.
(4) The proposed approach has good generality and can be applied to solving similar problems. The kinematic equation of bridge structure is established based on the general dynamic principle. Then, the nonlinear restoring force of the pier column bottom section is described by the Bouc–Wen model, and the nonlinear motion equation of the continuous girder bridge under multi-point seismic excitation is rewritten into a quasilinear equation, which is also established by combining with the Runge–Kutta method and a precise time-history integration method. The explicit dimension reduction iterative method in time domain adopted in this article is essentially a rapid method for solving local nonlinear random vibration of a class of problems, which is applicable as long as the problem can be described as a local nonlinear problem and contains an explicit nonlinear restoring force model. The above methods are not only appropriate to continuous girder bridges, but also applicable to the analysis of similar problems of other bridges.

## Figures and Tables

**Table 1:** Basic parameters of the bridge structure.

	Area (m^2^)	Moment of Inertia Iy (m^4^)	Moment of Inertia Iz (m^4^)	Elastic Modulus (GPa)	Density (kg/m^3^)
Girder	12.49	6.70	228.91	34.5	2549
Pier	2.01	0.32	0.32	32.5	2549

**Table 2:** Mander constitutive parameters of pier fiber section concrete.

Type (C40)	Elastic Modulus *E_c_* (MPa)	*f_c_* (MPa)	Yield Strain	Peak Strain	Ultimate Strain
non-confined concrete	31623	40.00	0.0014	——	0.02
confined concrete	31623	41.72	0.002429	0.002	0.013084

**Table 3:** Park constitutive parameters of pier fiber section steel.

Type	*E_s_* (MPa)	*f_y_* (MPa)	*f_u_* (MPa)	*ε_y_*	*ε_sh_*	*ε_su_*
HRB400	200,000	400	540	0.002	0.01	0.1

**Table 4:** Parameters of the Clough–Penzien spectrum.

Site Condition	$\omega_{k}$ (rad/s)	$\zeta_{k}$	$\omega_{sk}$ (rad/s)	$\zeta_{sk}$
Soil 1	20.94	0.6	1.5	0.6
Soil 2	10.0	0.4	1.0	0.6

**Table 5:** Time-consuming comparison of the two methods.

Method	Time (s)
explicit time-domain method	250
*Newmark-β* method	5000

**Table 6:** Statistical value of the bending moment at the bottom section of *P*2.

Average Value × 10^3^ (kN·m)	Average Value of the Standard Deviation × 10^3^ (kN·m)	Average Value of the Absolute Maximum × 10^3^ (kN·m)
1.89	6.22	18.04

## Data Availability

Not applicable.
